# Fast strain mapping in abdominal aortic aneurysm wall reveals heterogeneous patterns

**DOI:** 10.3389/fphys.2023.1163204

**Published:** 2023-06-08

**Authors:** Marta Irene Bracco, Magdalena Broda, Ulver Spangsberg Lorenzen, Mateusz Cezary Florkow, Oudom Somphone, Stephane Avril, Marco Evangelos Biancolini, Laurence Rouet

**Affiliations:** ^1^ Mines Saint-Étienne, University Jean Monnet, INSERM, Sainbiose, Saint-Étienne, France; ^2^ Philips Research Paris, Suresnes, France; ^3^ Department of Vascular Surgery, Rigshospitalet, Copenhagen, Denmark; ^4^ Department of Enterprise Engineering “Mario Lucertini”, University of Rome “Tor Vergata”, Rome, Italy

**Keywords:** abdominal aortic aneurysm, ultrasound B-mode cine-loops, vascular wall strains, strain imaging, ultrasound elastography, ultrasound simulations, radial basis functions, finite element modeling

## Abstract

Abdominal aortic aneurysm patients are regularly monitored to assess aneurysm development and risk of rupture. A preventive surgical procedure is recommended when the maximum aortic antero-posterior diameter, periodically assessed on two-dimensional abdominal ultrasound scans, reaches 5.5 mm. Although the maximum diameter criterion has limited ability to predict aneurysm rupture, no clinically relevant tool that could complement the current guidelines has emerged so far. *In vivo* cyclic strains in the aneurysm wall are related to the wall response to blood pressure pulse, and therefore, they can be linked to wall mechanical properties, which in turn contribute to determining the risk of rupture. This work aimed to enable biomechanical estimations in the aneurysm wall by providing a fast and semi-automatic method to post-process dynamic clinical ultrasound sequences and by mapping the cross-sectional strains on the B-mode image. Specifically, the Sparse Demons algorithm was employed to track the wall motion throughout multiple cardiac cycles. Then, the cyclic strains were mapped by means of radial basis function interpolation and differentiation. We applied our method to two-dimensional sequences from eight patients. The automatic part of the analysis took under 1.5 min per cardiac cycle. The tracking method was validated against simulated ultrasound sequences, and a maximum root mean square error of 0.22 mm was found. The strain was calculated both with our method and with the established finite-element method, and a very good agreement was found, with mean differences of one order of magnitude smaller than the image spatial resolution. Most patients exhibited a strain pattern that suggests interaction with the spine. To conclude, our method is a promising tool for investigating abdominal aortic aneurysm wall biomechanics as it can provide a fast and accurate measurement of the cyclic wall strains from clinical ultrasound sequences.

## 1 Introduction

An abdominal aortic aneurysm (AAA) is an irreversible dilation of the arterial wall usually localized in the infrarenal portion of the abdominal aorta, resulting in a balloon-like bulge. Mostly affecting the elderly male population, with a prevalence of between 4% and 8% in men aged above 65, it is often described as a silent killer since it becomes symptomatic only upon rupture (Nordon et al., 2010). Given the degenerative nature of AAA, regular surveillance is fundamental after diagnosis. As of today, a preventive surgical procedure is the only viable treatment if the bulge size reaches a certain threshold (Chaikof et al., 2018). Current guidelines prescribe the maximum diameter criterion as an independent risk factor for surgical decision-making. This criterion consists of a gender-adjusted threshold for surgery (i.e., 5 cm for women and 5.5 cm for men) and is based on large population studies (Moll et al., 2011). Despite the maximum diameter being an established criterion in clinical practice, serious concerns about its efficacy have emerged (Raut et al., 2013).

Alongside morphological assessment, several studies suggest that wall biomechanics provide important information on the degree of severity of the disease. Wall strain in response to blood pulse pressure is a measure of wall distensibility and can provide insight into disease progression (Wilson et al., 2003; Cebull et al., 2019). In addition, peak wall stress has been associated with rupture risk (Fillinger et al., 2003; Polzer et al., 2020). To correctly perform stress analyses, patient-specific material properties including wall stiffness are fundamental (Bruder et al., 2020). Material testing can provide a detailed characterization of the aortic tissue, but it can only be performed post-operatively. Conversely, *in vivo* estimations of AAA mechanical properties based on time-resolved AAA imaging could complement the current clinical guidelines. Specifically, AAA wall motion analysis allows for strain estimation, which in turn can be related to the wall stresses or can be used for inverse identification of material properties (Bihari et al., 2013).

In current clinical practice, the most reliable imaging technique to assess the AAA diameter is computed tomography, or, alternatively, the recently emerging three-dimensional ultrasound (3D US) imaging techniques (Ghulam et al., 2021). However, computed tomography provides a static representation of the AAA, while the low frame rate of 3D US (up to 20 Hz) limits its usability for deformation tracking. Conversely, two-dimensional ultrasound (2D US) B-mode images can be acquired at a high frame rate using a 2D probe to image a single plane (up to 80 Hz) or using a 3D probe to perform bi-plane acquisitions (up to 40 Hz) (Bihari et al., 2013; Wittek et al., 2017). Because of their high temporal resolution and widespread use in clinical practice, 2D US cine-loop sequences are viable candidates for clinical research studies on wall motion and for clinical applications with real-time measurements, overall contributing to the clinical translation of AAA non-invasive biomechanical assessment.

Based on 2D US, several tools have been developed to calculate the mechanical properties of the AAA wall. Assuming a circumferential vessel section, the cyclic AAA diameter variation was used as a measure of the global wall strain, as in the Diamove tool (Teltec, Lund, Sweden). The global wall stiffness was also estimated by relating the diameter change to blood pressure variation, as described in Hansen et al. (1993). Although very straightforward, this global method showed poor predictive power, suggesting the need for local wall strain estimation methods (Lorenzen et al., 2021).

In general, the steps needed to compute the AAA wall strains are segmentation, motion tracking, and displacement estimation. Segmentation is usually a manual delineation of the wall in the first frame of the sequence, which is then automatically tracked in the successive frames. To measure circumferential strains along the aortic wall, tracking algorithms were used to track the wall motion in 2D US cine-loops (Brekken et al., 2006; Vonk et al., 2014; Mix et al., 2017). In an early study (Brekken et al., 2006), the wall was segmented with a single layer of points. The wall motion along the tangential direction was accurately recorded for the selected points with M-mode imaging, but only on a single dimension. In later works, alongside the improvement in image quality and spatial resolution, 2D multi-layer strategies for AAA wall motion tracking were proposed by several authors. Their main goal was to analyze displacements in the wall thickness and, thus, provide deeper insight into the local wall strain. The tracking algorithms were based on cross-correlation between radio-frequency signals (Vonk et al., 2014; Mix et al., 2017; de Hoop et al., 2020). Alternatively, B-mode speckle tracking echocardiography was adapted for AAA 2D and 3D US sequences (Bihari et al., 2013; Derwich et al., 2020; Li et al., 2021). However, while single-layer methods are almost real-time, tracking wider areas of interest significantly increases the time needed for strain mapping, resulting in up to 60 min per scan (Zottola et al., 2022).

In addition to computational costs, previous studies have also reported critical challenges related to image quality for clinical translation of non-invasive AAA mechanical estimations (Petterson et al., 2021). B-mode image formation depends on the direction of acoustic scattering generated by the soft tissues. As a consequence, image quality can vary locally according to the position and orientation of the tissues. In particular, AAA lateral walls are hard to image because they are oriented parallel to the transmitted beams, thus producing a weaker echo scatter signal. It follows that tracking accuracy may vary along the circumference of the aortic wall, especially in lateral locations with lower echogenicity.

Once the wall motion is tracked, strains can be directly computed from the displacement field. A simple approach consists in computing the engineering strain: the wall contour is discretized in the circumferential direction, and the local strain is calculated as the ratio between the elongation and the initial length of each segment making up the wall (Brekken et al., 2006). The main limitation of this approach is that the strains are given only on a single dimension, i.e., the curvilinear coordinate describing the wall and, therefore, the strain estimation is highly dependent on segmentation and discretization methods. Another common method relies on the numerical differentiation of a dense 2D displacement field based on bilinear shape functions, the typical approach of the finite-element method (FEM), resulting in full-field strain representations (Mix et al., 2017; Zottola et al., 2022). However, this approach requires performing tracking on a regular mesh, constraining the location of the tracking points to the nodes of this mesh. To remove the need for a regular grid for computation, meshless methods, and specifically radial basis function (RBF)-based methods, can be employed to solve relatively simple mechanical problems with complex geometries from sparse source points (Patel and Rachchh, 2020).

In this work, we aimed to contribute to the clinical translation of AAA wall biomechanical estimations by proposing a fast, non-invasive, and accurate 2D strain mapping method based on clinical dynamic US acquisitions. By combining a sparse tracking algorithm and RBF strain computation, we designed a method that can adapt to the heterogeneous US image quality as well as deal with the time constraints typical of multi-layer approaches. In addition, we performed a feasibility study on a small cohort of patients, and we independently validated the tracking and strain computation algorithms.

## 2 Materials and methods

**FIGURE 1:**
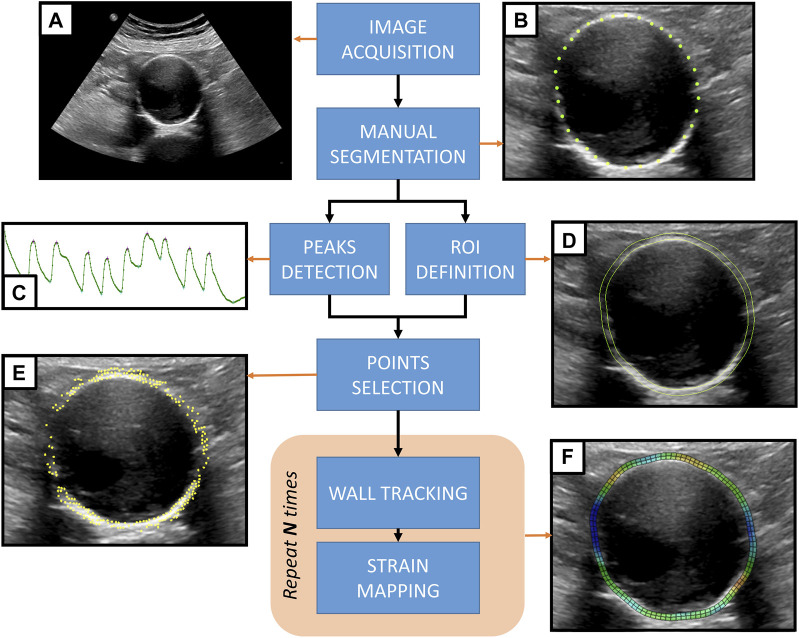
Proposed methodology for strain mapping in the AAA wall. **(A)** 2D US cine-loop sequence acquired from an AAA patient. **(B)** The AAA inner wall is manually segmented by placing landmark points on the first frame of the sequence. **(C)** Automatic motion tracking analyzes the evolution of the antero-posterior diameter throughout the sequence to detect the pressure cycle peaks. **(D)** A region of interest (ROI) is defined based on the manual segmentation of the wall. **(E)** An automatic point selection algorithm is applied in the region of interest, defining tracking points. **(F)** For each pressure cycle, the wall motion is tracked, and the strain maps are computed to be displayed as an overlay grid on top of the 2D US sequence.

The study pipeline can be summarized as follows. AAA patients were scanned in a clinical setting to acquire 2D US cine-loop sequences. Data were then transferred to an off-cart computer for processing. Subsequently, the inner wall of the vessel was manually segmented in the first frame of the sequence. Automatic tracking of the antero-posterior (AP) diameter was used to define cardiac cycles and select the most stable cycles. The rest of the procedure was fully automatic and consisted of an edge-based point selection algorithm that ran on the first frame of the first stable cycle. The selected points in the vessel wall were tracked along the cycles, and their displacements were computed. The strain corresponding to the displacements was then estimated and mapped on the B-mode image. The proposed methodology, including all steps required to generate AAA wall strain maps from transversal 2D US cine-loops, is presented in Figure 1. The remainder of this section details each step.

### 2.1 Data collection

2D US cine-loop sequences of 10 untreated AAA patients were acquired by trained medical doctors at Rigshospitalet Copenhagen in June 2021. The patients were enrolled in a study on AAA surveillance approved by the Danish Regional Research Ethics Committee (record number H-20001116) and gave their written consent. For each of the 10 patients, US B-mode cine-loop sequences were acquired during breath-holds in supine position with an EPIQ (Philips Healthcare) scanner. For each patient, we acquired a single-plane sequence with a 2D probe (C5-1) and a bi-plane sequence with a 3D probe (X6-1). The bi-plane sequences comprised both transversal and longitudinal views. For this study, we analyzed the transversal view. The image resolution ranged between 0.2 mm × 0.2 mm and 0.3 mm × 0.3 mm. Probes were positioned on the patient’s abdomen in order to visualize the maximum AP AAA diameter section in the transversal plane according to clinical guidelines. In this orientation, the posterior wall of the AAA is at the bottom of the scan, anatomically adjacent to the vertebral column, whereas the anterior wall is at the top, on the probe side. As in standard AAA echography, the patient’s anatomic left is observed on the right of the scan view. The US focal depth was placed adjacent to the posterior AAA wall, at the AAA–vertebra interface. No contrast medium was to be present in the patient’s blood. Two patients were excluded from the analysis: one had contrast medium in their bloodstream and the other displayed large breathing artifacts and low image quality. As a result, data from eight patients were deemed suitable for the analysis.

### 2.2 Inner wall segmentation

The 2D US sequences were post-processed offline in non-commercial prototype software based on the Visualization Toolkit (Schroeder et al., 1998). The internal wall perimeter was manually segmented by placing landmark points where the interface profile was clear (Figure 1B). Landmark placement was more challenging in the lateral walls due to the lack of echoes, and, when visible, at the interface with the intraluminal thrombus, which is more echogenic than blood. The final segmentation of the inner wall was achieved by interpolating the points and discretizing the contour to a fixed, regularly spaced number of points.

### 2.3 Region of interest and output grid

**FIGURE 2:**
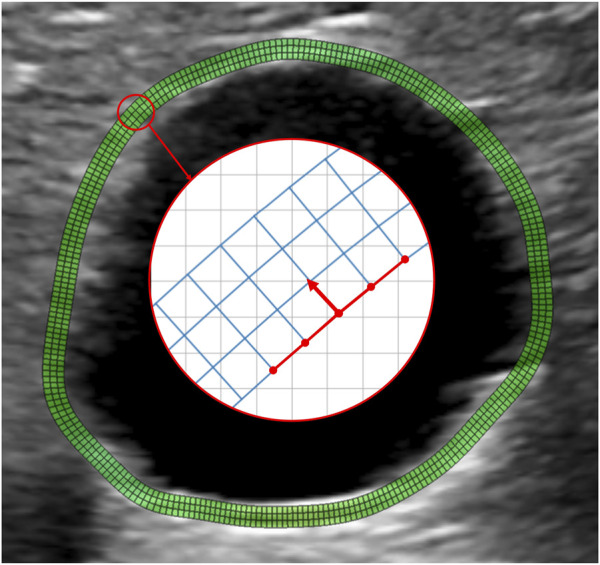
Example of a wall grid used for strain mapping. The grid is obtained by resampling the manually selected contour with N equally spaced points. Each of the N points is projected outward along the normal direction at a distance of 2 mm from the inner wall. The normal direction is obtained by a 90° rotation of the segments connecting the point to the four adjacent grid nodes, as shown by the red arrow. The obtained outer wall is resampled again with N points to guarantee regular grid spacing, keeping the most posterior point fixed. The final grid is obtained by building two intermediate layers by partitioning the segment connecting the inner layer to the outer layer nodes into three segments. Therefore, the connecting segments are not necessarily normal to the vessel wall. N is chosen to guarantee an aspect ratio of the grid elements close to 1.

Since AAA wall thickness cannot be estimated from the US images, a standard homogeneous thickness of 2 mm was imposed. To define the region of interest (ROI) corresponding to the AAA wall, the points on the inner wall were projected by 2 mm in the normal direction to create the outer layer (Figure 1D). A uniformly spaced output grid was then generated for strain visualization using the inner and outer layers of the ROI. Figure 2 shows an example of an output grid with four layers. The grid density was determined in accordance with the results of the convergence study presented in Section 2.5.5.

### 2.4 Wall tracking algorithm

#### 2.4.1 Sparse Demons tracking

The Sparse Demons (SD) tracking algorithm was developed by Somphone et al. (2013) for the evaluation of regional myocardial function from high frame rate echocardiography sequences. SD compares well to other available methods in terms of accuracy, but is significantly faster, making it suitable for real-time applications (De Craene et al., 2013; Alessandrini et al., 2016). Frame-to-frame tracking is implemented by minimizing an energy function only on a predefined set of points, in order to find the best matching pixel in the reference image. A Gaussian model of the motion is also adopted so that the resulting displacement field is inherently smooth. Due to its sparse nature, the accuracy of SD is highly dependent on landmark selection. It follows that the tracking point location needs to be optimized according to local image quality, maximizing the extraction of motion information from the US sequences.

#### 2.4.2 Cardiac cycle peak detection

Using the SD algorithm, inner wall landmarks are tracked over time, using frame-to-frame tracking (forward and backward) on the whole sequence. The AP diameters are then measured at the intersection of the AAA wall contour and the vertical line crossing the center of the AAA for each frame. Local extrema in the AP diameter temporal evolution define the peak-systole (maxima) and end-diastole (minima) frames (Figure 1C). Notably, in this work, we adopted the typical cardiac terminology to refer to the abdominal aortic pressure cycle for simplicity, but a physiological delay exists. Each sequence can thus be split into pressure cycles starting at the beginning of systole (or end of the previous diastole). For each sequence, a cycle of reference is also selected as the cycle where the diameter variation from end-diastole to the next peak-systole is closest to the mean diameter variation. The peak detection algorithm was tested by determining the variability of the cycle length within a sequence as well as the AP diameter change.

#### 2.4.3 Sequence cropping

The default duration of acquisition was set to 10 s in order to maximize the chance of observing multiple complete regular pressure cycles. However, a breath-hold of such a long duration can be challenging to achieve in clinical practice, especially for elderly patients. As a consequence, most of the sequences exhibited a few cycles with motion artifacts due to breathing or abdominal muscle contractions. Therefore, visual inspection of the temporal curves of the AP diameter was performed to select sub-sequences without motion artifacts. Cropped sequences were used for further strain computation. Sequence cropping was performed manually, leaving at least two consecutive cycles per patient.

#### 2.4.4 Automatic selection of tracking points

To deal with the heterogeneity of the B-mode image quality and only track the points in the wall where a speckle is visible, an automatic algorithm for tracking point selection was developed. The points were selected on the first tracked frame, i.e., the start of the first systole of the sequence. First, the gradient of the image was calculated as the sum in two dimensions of the Sobel operator (Sobel and Feldman, 1968). A sorting algorithm was then implemented to select the points based on their gradient value. To avoid clustering, a minimum distance between the points was imposed. This distance constrains the number of points in an image and, thus, the computational cost, and it was empirically set to 1 mm as no improvement was observed for smaller distances. The points were selected only within and on the edges of the ROI (Figure 1E). Thanks to the SD algorithm, we could only track the selected points and extract the most relevant motion patterns in the image.

#### 2.4.5 AAA wall tracking

The SD tracking algorithm was applied to the set of selected points and provided for each point of the frame-to-frame displacement values. Each pressure cycle was tracked independently by running the algorithm once forward and once backward within the cycle frame range. The resulting values of displacement were computed by merging the results from the forward and backward procedures.

#### 2.4.6 Tracking validation with simulated US sequences

Validation of the tracking algorithm was previously performed by Somphone et al. (2013) for myocardial strain mapping. We propose a similar validation strategy for the AAA wall motion tracking. Specifically, we have simulated eight 2D US sequences for which the displacement field is known. In order to achieve realistic simulation, the MATLAB Ultrasound Simulation Toolbox (MUST, Garcia (2021)) was employed. MUST allows the generation of a B-mode image starting from a template image. Acoustic echoes are simulated by convolving an acoustic pressure field with the impulse response of a set of independent monopole point sources, or scatterers, in the frequency domain. Each scatterer generates an echo proportional to its reflection or backscattering coefficient (BSC). The BSC was calculated based on the intensity level in the corresponding location on the template image, as explained by Alessandrini et al. (2015). For a generic scatterer *s*, we used the following relationship

$${BSC}_{\text{s}}=\frac{I_{\text{s}}}{255\gamma },$$ (1)

where ***I***_***s***_ the pixel amplitude and *γ* is an experimental parameter fixed to 0.2. The scatterers were selected on the image domain in random locations, and their density was set experimentally as a compromise between image quality and the computational cost of the simulation, resulting in around 11,000 scatterers per frame.

To simulate the displacement of the wall, 2D US cine-loop sequences obtained from the patients were used as templates. From each sequence, the frames belonging to the first detected cycle were extracted. To capture the motion, we adopted the approach proposed by Alessandrini et al. (2018): the scatterers were grouped into two distinct maps, the moving (coherent) map and the background (incoherent) map, depending on their location. The scatterers inside the wall ROI were in the coherent map and were assigned fixed BSC values in the first frame of the template sequence. Conversely, the scatterers in the background were in the incoherent map, and their BSC was updated at each frame. While the location of the incoherent scatter map was fixed, the coherent maps moved according to a predetermined displacement field. Finally, a transition zone of 1 mm between the two maps was created. The wall displacement field was generated by exporting the sequence output grid to a FEM solver (ABAQUS Inc.) and simulating a plane strain inflation test with a uniform pressure of 3 kPa, followed by deflation to the initial state. The inflation and deflation phases were matched to the systolic and diastolic phases in the sequence, respectively, and the output of the simulation was sampled with the sequence frame rate. In this way, we obtained a temporal coherence between the real sequence and the simulated one. The nodal displacements resulting from the FEM simulation were considered the ground truth, and they were applied to the coherent scatter map after interpolation. The simulation of a single B-mode 2D US sequence took 224 min for a 33-frame cycle using 20 parallel threads on a 2 CPU Intel Xeon 10 core 2.6 GHz processor.

For validation, the nodes of the output grid, used as an input to the FEM, were placed on the first frame of the sequence (i.e., the beginning of the first cycle) and tracked with the SD algorithm. The obtained displacement field was then compared to the ground truth to evaluate the tracking accuracy. We computed the error in each frame and each point of the grid as the distance between the tracked points and the corresponding ground truth points to have a local measure of the error. The proposed method was also used to test the benefits of automatic point selection. Each sequence was tracked both using the output grid and the automatic segmentation approach. Then, we computed the root mean square error (RMSE) in all frames.

### 2.5 Strain computation

#### 2.5.1 RBF interpolation

Frame-to-frame tracking results in a sparse displacement distribution. As a further step, to obtain a continuous differentiable displacement field, the discrete field was interpolated with respect to the first frame of the tracked cycle. This interpolation served as a first step toward the strain computation (1 F). For this purpose, RBF interpolation and differentiation were adopted to accurately map AAA wall strains, similar to what was performed by Biancolini (2017) and Chiappa et al. (2019) for upscaling FEM results and by Dai et al. (2015) and Groth et al. (2022) for digital image correlation. Specifically, we leveraged the meshless property of RBF, meaning it does not require a regular grid, allowing the computation of strains directly from the sparse point cloud used for tracking.

RBFs are a class of interpolator functions capable of building multi-dimensional fields starting from sparsely distributed source points, or point clouds. The value of the RBF field in each point depends on its weighted distance (or radius) to the rest of the source points. In our study, we have used a cubic RBF, for which we can write, given N source points **x**_***i***_, the displacement value at a target point x of coordinates (*x*, *y*) as

$$u\left(x\right)=\overset{N}{\underset{i=1}{\sum }}\gamma_{i}{\left(\left\|x-x_{i}\right\|\right)}^{3}+h\left(x\right),$$ (2)

where $\left\|x-x_{i}\right\|$ is the Euclidean distance between the target and one source, *γ*_*i*_ contains the RBF weights, and **h** is the polynomial term, expressed in 2D as

$$h\left(x\right)=\beta_{0}+\beta_{1}x+\beta_{2}y.$$ (3)

To find the weights *γ*_*i*_ and the polynomial coefficients *β*_*i*_, we solved a linear system, once for *x* and once for *y* at known displacement values, i.e., in the source points.

$$\left[\begin{array}{cc} M & P\\ P^{T} & 0 \end{array}\right]\left(\begin{array}{c} \gamma \\ \beta \end{array}\right)=\left(\begin{array}{c} g\\ 0 \end{array}\right),$$ (4)

where **g** is the vector containing the known values of displacement and **M** is the interpolation matrix, given by, in index notation:

$$M_{ij}={\left(\left\|x_{i}-x_{j}\right\|\right)}^{3},$$ (5)

**P** is the constraint matrix from the orthogonality condition imposed by the polynomial term, expressed in 2D as

$$P=\left[\begin{array}{ccc} 1 & x_{1} & y_{1}\\ 1 & x_{2} & y_{2}\\ \vdots & \vdots & \vdots \\ 1 & x_{N} & y_{N} \end{array}\right].$$ (6)

In our method, we employ a linear solver using the common LAPACK routine for numerical algebra.

Once the coefficients were found, we obtained two continuous displacement fields, one in each dimension, which can be written as

$$\left\{\begin{array}{c} u^{x}\left(x\right)=\sum_{i=1}^{N}\gamma_{i}^{x}{\left(\left\|x-x_{i}\right\|\right)}^{3}+h^{x}\left(x\right)\;\\ u^{y}\left(x\right)=\overset{N}{\underset{i=1}{\sum }}\gamma_{i}^{y}{\left(\left\|x-x_{i}\right\|\right)}^{3}+h^{y}\left(x\right).\; \end{array}\right.$$ (7)

Finally, we calculated the values of displacement in the output grid nodes for plotting.

#### 2.5.2 RBF differentiation

The displacement fields in 7 were differentiated to express an analytical 2D strain field. First, the partial derivatives of the displacement field were calculated to obtain the material displacement gradient tensor **∇**_**x**_ **u**, given in 2D by

$$\nabla_{x}u=\left[\begin{array}{cc} \frac{\delta u^{x}}{\delta x} & \frac{\delta u^{x}}{\delta y}\\ \frac{\delta u^{y}}{\delta x} & \frac{\delta u^{y}}{\delta y}. \end{array}\right]$$ (8)

The expression for the gradient **∇**_**x**_ **u** can be found by applying the chain rule for differentiation to 7. To illustrate the process, we write the first term of **∇**_**x**_ **u**

$$\frac{\delta u^{x}}{\delta x}=\overset{N}{\underset{i=1}{\sum }}\gamma_{i}^{x}3{\left(\left\|x-x_{i}\right\|\right)}^{2}\cdot \frac{\left(x-x_{i}\right)}{\left\|x-x_{i}\right\|}+\beta_{1}.$$ (9)

Applying the infinitesimal strain theory, we can write the expressions for the normal strain in *x* and *y* directions as

$$\varepsilon_{x}=\frac{\delta u_{x}}{\delta x},\varepsilon_{y}=\frac{\delta u_{y}}{\delta y},$$ (10)

and that for the engineering shear strain as

$$\gamma_{xy}=\gamma_{yx}=\frac{\delta u_{x}}{\delta x}+\frac{\delta u_{y}}{\delta y}.$$ (11)

#### 2.5.3 Strain mapping on local polar coordinates

To obtain a clinically interpretable mapping following a local polar coordinate system, we extrapolated the values of displacements and strains in the output grid. For each point on the grid, we calculated the corresponding tangent vector *t* and the angle *θ* spanned by t with respect to the horizontal axis, as depicted in Figure 2. The rotation matrix **T** was then calculated for each point on the grid as

$$T=\left[\begin{array}{ccc} m^{2} & n^{2} & 2mn\\ n^{2} & m^{2} & -2mn\\ -mn & mn & m^{2}-n^{2} \end{array}\right]\;with\left\{\begin{array}{c} m=\cos\left(\theta \right)\;\\ n=\sin\left(\theta \right),\; \end{array}\right.$$ (12)

and applied to the Cartesian coordinates

$$\left[\begin{array}{c} \varepsilon_{\mathrm{circ}}\\ \varepsilon_{\mathrm{rad}}\\ \frac{\varepsilon_{\mathrm{shear}}}{2} \end{array}\right]=T\cdot \left[\begin{array}{c} \varepsilon_{x}\\ \varepsilon_{y}\\ \frac{\gamma_{xy}}{2} \end{array}\right],$$ (13)

where *ɛ*_*circ*_ is the circumferential strain, tangent to the grid; *ɛ*_*rad*_ is the radial strain, normal to the grid; and *ɛ*_*shear*_ is the shear component with respect to the grid.

#### 2.5.4 Inter-cycle and inter-probe reproducibility analysis

Circumferential, radial, and shear strains were evaluated in the systolic frames of each sequence in order to evaluate their reproducibility from one cycle to another. The interclass correlation coefficient (ICC) was used to estimate the absolute agreement between the strain measurements in different cycles. This coefficient, ranging from 0 to 1, is calculated by applying a two-way random-effect model and can be interpreted as good when above 0.75 and poor when below 0.5, as described in Koo and Li (2016). The inter-probe reproducibility was visually assessed in terms of a sector-wise comparison of the wall strains in the circumferential direction.

#### 2.5.5 Strain validation and comparison with FEM

**FIGURE 3:**
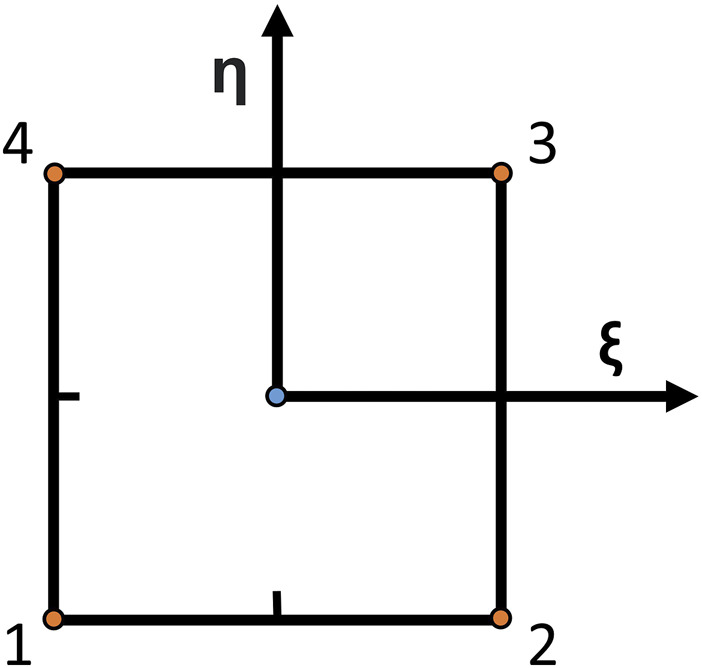
Schematic of a bilinear element with reduced integration. Linear elements approximate the finite-element method solution and are very common as they are computationally efficient and robust to large element distortions, simplifying the meshing problem. They have four nodes (orange dots), and the solution is calculated in the element centroid (blue dot). *ξ* and *η* are the directions from which shape functions are defined.

To validate our strain calculation method against an established one and assess its performance, we compared the nodal strains in the output grid obtained with our RBF-based approach to the nodal strains calculated with the bilinear shape function method, typical of the FEM method. The grid was interpreted as a mesh by the finite-element solver, and the displacements were assigned as boundary conditions to the nodes. The analysis was performed with ABAQUS 2021 (Dassault Systems Simulia Corp., France). For the FEM problem, the domain was discretized with plane strain linear elements. For each element, the strain is computed most accurately in the element’s integration point, which, for the chosen element type, corresponds to the centroid Barlow (1976). The chosen elements have a reduced integration scheme and were chosen because of their widespread use in engineering applications. They are briefly described in Figure 3. To compare the results of our method to the FEM results, we extrapolated the maximum principal strain values at the same locations.

We repeated the same experiment using various grid densities to study the mesh convergence of the two methods, starting from a size of 2 mm down to the image resolution. For each case, we also calculated the convergence of the two methods by subtracting the strain values in the Cartesian coordinates for each mesh refinement.

## 3 Results

### 3.1 Cardiac cycle peak detection

**FIGURE 4:**
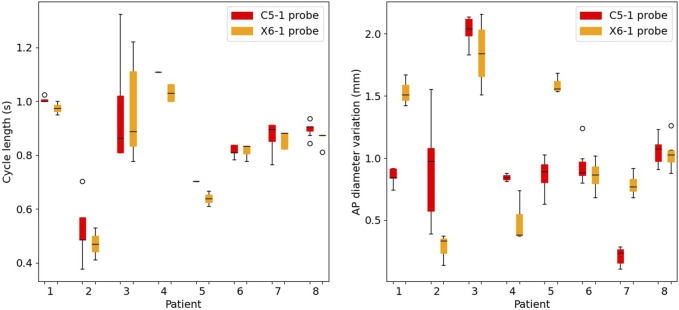
Boxplots of the cycle length (left) and of the cyclic antero-posterior diameter variation (right) in the selected cycles for all patients. For each patient, results are presented for the C5-1 probe (red) and X6-1 probe (orange).

On average, five consecutive cycles were analyzed in each sequence. The performance of the peak detection algorithm is presented in Figure 4. The inter-cycle variability of cycle length and the AP diameter change are shown for both probes (C5-1 and X6-1). The pressure cycle duration among the patients ranged between 0.37 s and 1.69 s. The AP diameter variation between start and peak-systole ranged between 0.24 mm and 2.14 mm.

### 3.2 Tracking

**FIGURE 5:**
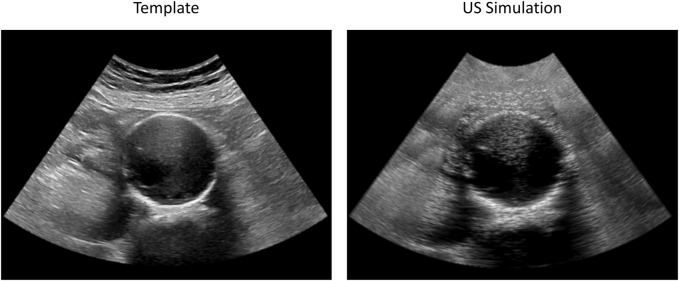
Examples of original (left) and simulated (right) 2D US frames used for AAA wall tracking validation. The original sequence (Patient 3) was acquired with a C5-1 probe. Both images have a spatial resolution of 0.22 mm × 0.22 mm. The animated simulated sequence is reported in Supplementary Video S1.

**FIGURE 6:**
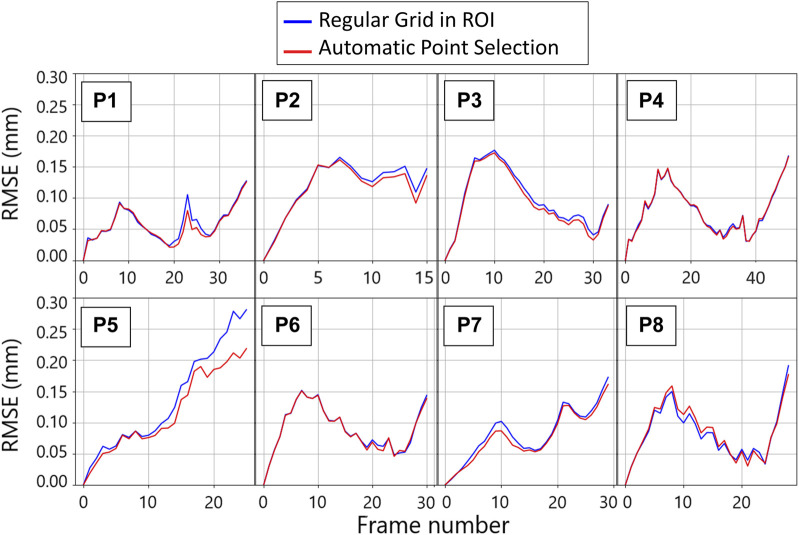
Per patient evolution of the root mean square error (RMSE) between the imposed ground truth displacement field and the tracking result for two tracking strategies throughout the first cycle of the template sequence. The blue plots are obtained by tracking the wall in the points corresponding to the nodes of the regular grid defined for strain mapping. The red lines are obtained by tracking the points that were automatically selected based on the gradient values.

The tracking algorithm was validated against simulated 2D US sequences, where the AAA wall was displaced according to a physical simulation of the blood pressure in a cycle. We report an example of a frame of these simulations in Figure 5 together with the template image. An animated version of the sequence is provided in Supplementary Video S1. Figure 6 shows the RMSE for each considered sequence and compares the performance of the tracking method with and without applying the automatic point selection algorithm. In all patients but one (P8), the automated point selection proved better than that of the regular grid. The highest reported error was 0.28 mm, up from 0.22 mm when the automatic tracking algorithm was used.

### 3.3 Strain mapping

**FIGURE 7:**
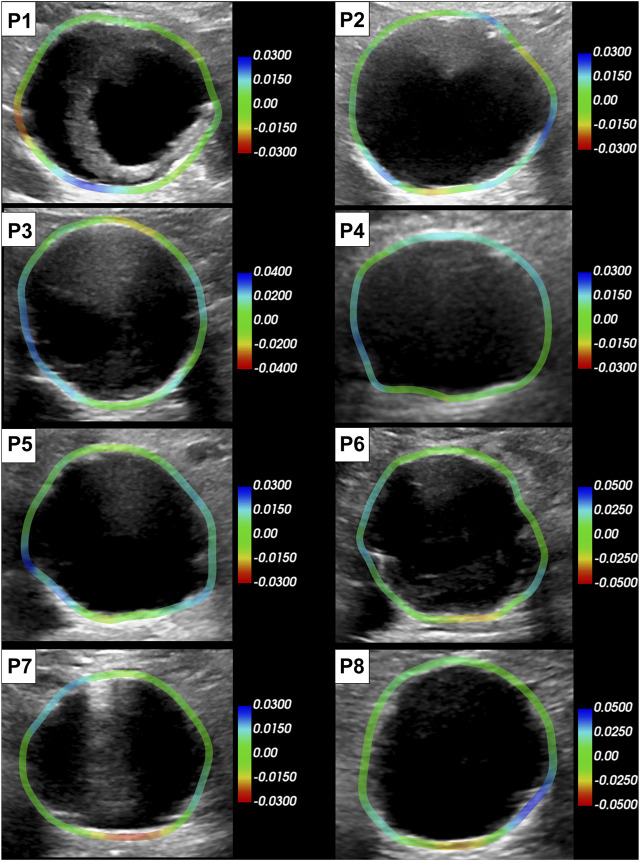
Circumferential strain maps overlaid on the reference peak systolic frame of each patient (C5-1 probe). The color scale is adapted per patient based on the range of the strain in the sequence. Positive strains (blue) indicate circumferential stretch and negative (red) indicate circumferential shortening.

**FIGURE 8:**
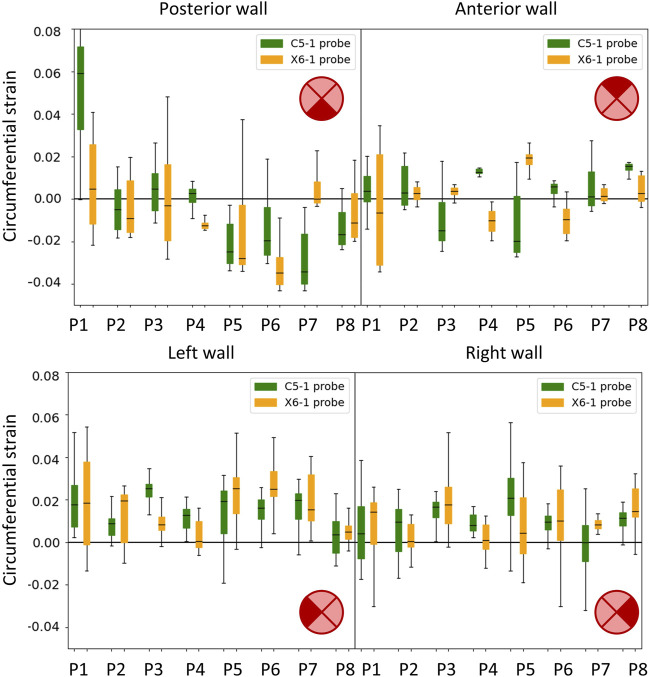
Boxplots showing the circumferential strains in the cycle of reference. The wall was divided into four sectors: posterior (top left), anterior (top right), left side (bottom left), and right side (bottom right) in accordance with the transversal 2D US imaging plane. Results are presented for the C5-1 probe (green) and the X6-1 probe (orange).

**FIGURE 9:**
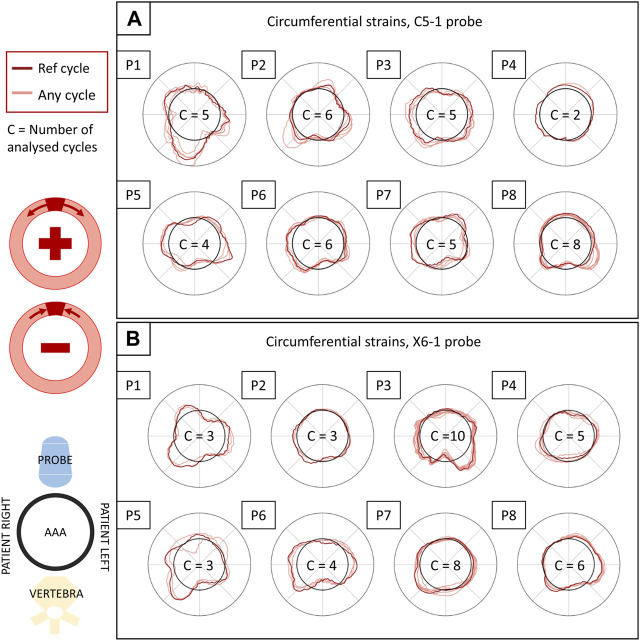
Circumferential strains in all patients and their inter-cycle reproducibility, reported for the C5-1 probe **(A)** and the X6-1 probe **(B)**. The polar plots depict the circumferential strains in the systolic peaks of each analyzed cycle (in light red) and in the reference cycle (in dark red), averaged over the layers of the output grid. The representation is coherent with 2D US orientation. For each sequence, the number of analyzed cycles is reported inside the polar plot. The strain values (radial coordinate in the plots) range from −0.01 to 0.01. The meaning of negative and positive values is illustrated in the scheme on the left.

**FIGURE 10:**
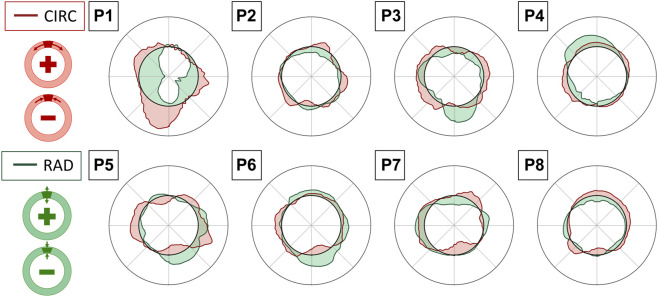
Superimposition of circumferential (CIRC-red) and radial (RAD-green) strain polar plots in the reference cycle peak systolic frame, averaged over the layers of the output grid. Interpretation of positive and negative values in circumferential (top) and radial (bottom) directions is provided in the schemes on the left.

All the single-plane and bi-planar sequences were processed to map the wall strains in the transverse plane. Figure 7 reports the circumferential strain maps from all the sequences acquired with the C5-1 probe. For this representation, we show the results on the peak systolic frame in the reference cycle, selected in the peak detection phase. The full animated sequence with overlay for P8 is reported in Supplementary Video S2 along with the X6-1 probe map S1. As illustrated, negative circumferential strains indicate a decrease in length and positive values indicate stretch. Overall, the circumferential strains ranged between −0.04 and 0.08. Figure 8 shows the sector-wise mean circumferential strains in the reference frame for each patient and probe. All patients, except P1, exhibit negative (P2 and P5 to P8) or zero strain (P3 and P4) in the posterior wall, as well as circumferential stretch in the lateral sides. Anterior wall strain is always close to zero, except in P3, where it is negative, and P2, where it is positive. The circumferential strain distributions in all patients can be assessed as shown in Figure 9 for both the C5-1 probe and the X6-1 probe. Radial and shear strain distributions are reported in Supplementary Figures S2, S3. In order to assess the relationship between circumferential and radial strains, Figure 10 reports a superimposition of the circumferential and radial strain maps for the C5-1 probe. Positive radial strains indicate wall thickening, while negative values indicate thinning.

The total computation time for each sequence is reported together with the time per cycle in Table 1 and the average number of frames per cycle. The computations were performed on a personal computer with an Intel (R) Core (TM) processor (i5-10310U CPU at 1.70 GHz).

**TABLE 1:** Computational time required to complete the analysis (excluding manual segmentation) and time to analyze a single cycle and an average number of frames per cycle.

Probe		1	2	3	4	5	6	7	8
C5-1	Analysis time (s)	298	278	220	173	109	250	182	289
	Time per cycle (s)	59.6	46.3	44.0	86.5	27.2	41.7	36.4	36.1
	Frames per cycle	34	17	14	50	19	32	25	26
X6-1	Analysis time (s)	89	70	263	148	53	90	173	93
	Time per cycle (s)	29.7	23.3	26.3	29.6	17.7	22.5	21.6	15.5
	Frames per cycle	15	8	13	14	7	12	13	13

### 3.4 Inter-cycle reproducibility

The inter-cycle reproducibility of strain measurements can be qualitatively assessed as shown in Figure 9. For each patient, peak systolic strains in all analyzed cycles are reported in a polar plot in the circumferential direction.

A quantitative measure of reproducibility is provided in Table 2, which reports the absolute ICC computed on the sequence’s peak systolic frames for all analyzed patients and probes. The ICC for the 2D probe was higher overall, with only two cases of poor reproducibility out of 24, while the bi-axial probe showed less reliable measurements, with seven cases of poor reproducibility out of 24.

**TABLE 2:** Intraclass coefficient as a measure of the inter-cycle reproducibility obtained for both probes. Red and ^(−)^ indicate poor reproducibility; green and ^(+)^ indicate excellent reproducibility (Koo and Li, 2016).

Probe	Direction	1	2	3	4	5	6	7	8
C5-1	Circular	$0.75^{(+)}$	$0.48^{(-)}$	0.71	$0.77^{(+)}$	$0.81^{(+)}$	$0.85^{(+)}$	0.72	$0.79^{(+)}$
	Radial	$0.81^{(+)}$	0.60	0.59	$0.99^{(+)}$	$0.87^{(+)}$	$0.76^{(+)}$	0.50	$0.78^{(+)}$
	Shear	$0.90^{(+)}$	0.69	$0.89^{(+)}$	$0.94^{(+)}$	0.72	$0.86^{(+)}$	$0.40^{(-)}$	$0.84^{(+)}$
X6-1	Circular	$0.91^{(+)}$	$0.49^{(-)}$	$0.86^{(+)}$	0.52	0.57	$0.90^{(+)}$	0.66	$0.92^{(+)}$
	Radial	0.73	$0.14^{(-)}$	0.74	$0.20^{(-)}$	$0.44^{(-)}$	0.67	$0.28^{(-)}$	0.65
	Shear	$0.94^{(+)}$	$0.25^{(-)}$	$0.88^{(+)}$	$0.41^{(-)}$	0.73	0.72	0.51	$0.88^{(+)}$

#### 3.4.1 Inter-probe reproducibility

**FIGURE 11:**
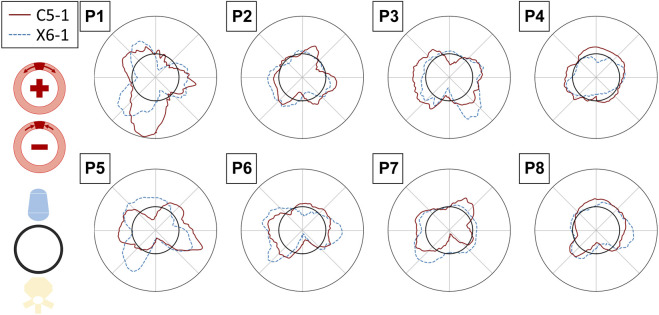
Polar plots assessing the inter-probe reproducibility of strain measures. The circumferential strains in the reference peak systolic frame are depicted for all the patients, averaged over the layers of the output grid, for the C5-1 probe (solid red line) and X6-1 probe (dashed blue line). The strain value (radial coordinate) ranges from −0.01 to 0.01. The polar plots have the same orientation as the 2D US scan (i.e., with the AAA posterior wall on the bottom).

A qualitative representation of the inter-probe variability is provided in the polar plots in Figure 11, where the circumferential strains are reported for each patient and probe in the peak systolic frame of reference. A visual assessment of inter-probe reproducibility reveals similar strain patterns in both probes, particularly in P3, P5, P6, and P8. The boxplots in Figure 8 quantify the sector-wise inter-probe variability of circumferential strain. The wall is split into four sectors depending on their location, as indicated by the schematics on the top right of each plot. Sector-wise comparisons showed good overall reproducibility in most sequences.

#### 3.4.2 Comparison with FEM integration

**FIGURE 12:**
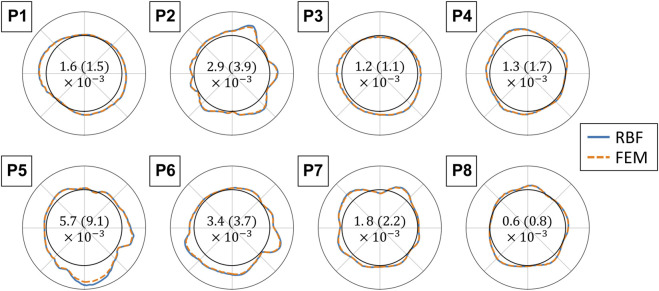
Polar plots comparing the finite element (FEM) and radial basis function (RBF) methods for strain validation. The maximum principal strains are displayed for each analyzed sequence with the RBF differentiation method (blue solid line) and with FEM shape functions (orange dotted line). The polar plots are in the same orientation as the 2D US scan, and the strain values range from 0 to 0.25 (radial coordinate). The values are reported for grids with an element size of 1 px and for the frame with the highest absolute difference. The mean absolute difference between the two methods and the standard deviation for each sequence are reported in the center of each polar plot (Mean (standard deviation)).

The polar plots in Figure 12 report the strains in the maximum principal direction as calculated by RBF and by FEM shape functions. For each case, the represented frame is the one where the maximum difference was found. The strains range from 0 to 0.25. The mean absolute difference between the strain values ranges between 0.6 × 10^−3^ and 5.7 × 10^−3^.

**FIGURE 13:**
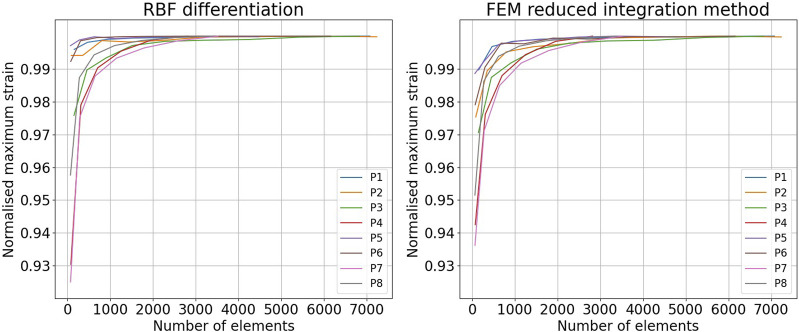
Comparison of the mesh convergence rates in the finite element method (FEM) and the radial basis function (RBF) methods. Convergence of the maximum strain in the maximum principal direction obtained with the RBF differentiation method (left) and the FEM reduced integration method (right).

Figure 13 shows mesh convergence curves of the maximum strain values (in maximum principal direction), normalized with respect to the convergence value, for the RBF differentiation method and for the FEM reduced integration method. The convergence rate of each sequence is shown. For all of the cases, the convergence of the RBF method is above 99% for a number of elements superior to 1,000, corresponding to around three element layers (depending on the aneurysm size). The convergence of the FEM method is slightly slower but still in the same range of values for the same number of elements.

## 4 Discussion

AAA rupture risk assessment is a multi-factorial problem, and although clear links with wall tissue biomechanics were found, translating these findings into clinical practice is still an open challenge (Vorp and Vande Geest, 2005; Bruder et al., 2020). Wall distensibility in response to pulsating blood pressure can provide clinicians with relevant information on the degree of wall degeneration, therefore contributing to decision-making (Wilson et al., 2003). However, the estimation of AAA wall biomechanical properties in the clinic is hindered by the lack of non-invasive measurement tools. Current methods make strong assumptions to reduce the computational burden (Hansen et al., 1993) but with unsatisfactory results in terms of risk assessment (Lorenzen et al., 2021). Previous studies have shown that it is possible to calculate the local strains in the AAA wall from 2D US data. Brekken et al. (2006) developed an almost real-time semi-automatic procedure. For computational purposes, the analysis was based on a single dimension (circumferential), which can only provide a partial representation of the wall biomechanics, as it neglects wall thickness (radial and shear). In addition, because they are only projected on a single layer, the resulting strains are highly dependent on the initial point selection. Two-dimensional, multi-layer approaches, like the one developed by Mix et al. (2017), provide a deeper insight into the wall tissue mechanical properties but are limited by their computational burden, reported between 30 and 60 min per sequence, including scan time. (Zottola et al., 2022)

To overcome the time constraints of the current methods while looking into a 2D strain field, we developed a fast multi-layer post-processing method for local *in vivo* strain mapping in the AAA wall from 2D US data. Our method requires an initial rough delineation of the AAA wall and, in some cases, a temporal cropping of the sequence. Then, the sequence is automatically analyzed, and 2D strain is mapped for all detected cardiac cycles.

### 4.1 Overall method performance

Our feasibility study showed promising results on a set of 16 2D US cine-loop sequences from eight patients, which were acquired in accordance with standard clinical practice. The automatic part of the method took between 15.5 s and 86.5 s per cycle on a personal computer (i5-10310U CPU at 1.70 GHz), depending on the cycle length and number of tracking points. For comparison, we allow 15–60 s for the manual segmentation and 10–30 s for the sequence cropping, depending on the complexity of the case and the operator’s expertise. The acquisition time should not take more than 1 min. Thus, considering that the longest sequence took 298 s, we expect the whole pipeline to take less than 8 min.

### 4.2 Cycle peak detection

Ten-second acquisitions allowed us to isolate at least ten cycles per sequence, automatically detected by tracking the AP diameter evolution throughout the sequence. Automatic cycle detection based on AP diameter evolution has been proven to be a robust strategy, avoiding relying on the simultaneous acquisition of the patient’s electrocardiogram (Brekken et al., 2006) or on visual assessment of the wall motion (Zottola et al., 2022). As long breath-holds and stillness during the acquisition are often challenging, undesired motion can emerge. Thus, manual cropping of the sequence was performed by the reader by looking at the AP evolution curve. At least two consecutive stable cycles per sequence were selected. After the manual sequence cropping was performed, the peak detection algorithm showed an inter-cycle variability of cycle length up to 0.4 s. The cardiac cycle duration was coherent between the two probes, and the cases of large rhythm variability were captured by both probes (e.g., P3 in Figure 4). In general, the X6-1 probe detected shorter cycles, which could be due to its lower temporal resolution. A cyclic AP diameter expansion of up to 2.1 mm was found, in line with the clinical assessments in Grøndal et al. (2012). In some cases (P1, P5, and P7), the two probes revealed a high difference in the values of AP diameter variation. This variability could be due to a difference in transducer position often associated with tortuous AAA Schäberle et al. (2015). An intra-probe reproducibility study is needed to confirm this hypothesis.

### 4.3 AAA wall tracking validation

The SD tracking algorithm allowed us to freely select the tracking points to adapt to the image quality Somphone et al. (2013). Specifically, in this study, an automatic procedure was developed to only select the points in the ROI where the gradient was above an empirically chosen threshold. As a consequence, it can be postulated that the manual wall segmentation had a limited effect on the tracking results, as it only influenced the ROI selection. SD tracking performance was previously validated on myocardial sequences (Somphone et al., 2013), finding a maximum global displacement error of 0.8 mm. In the present study, we proposed a validation strategy for AAA wall tracking based on physical simulations, adapting the pipeline presented in Alessandrini et al. (2018). Specifically, we simulated 2D US sequences of AAA with an imposed wall motion, which was used to evaluate the tracking algorithm accuracy. For most cases, the highest tracking errors were observed at systole, where tissue velocity is highest. However, for one case, (P5), the error followed a different pattern and kept increasing throughout the sequence. For this case, the high tracking error was associated with a signal void in the lateral wall where the tissue was not visible, and, therefore, could not be tracked. In general, the RMSE found was below 0.22 mm, which is within the B-mode spatial resolution. The automatic selection showed an improvement of up to 0.06 mm in the tracking performance compared to tracking on a regularly spaced grid. Thus, the results of our analysis indicate that selecting points based on the image gradient can better extract motion information from the 2D US sequence. The advantage of using an automated point selection based on gradient, as opposed to using a regular grid, was best demonstrated in cases with low-signal regions. In these regions, extrapolating motion from the adjacent wall segment using RBF fields proved more effective than tracking. In general, the RMSE found was below 0.22 mm, which is within the B-mode spatial resolution. The automatic selection showed an improvement of up to 0.06 mm in the tracking performance compared to tracking on a regularly spaced grid. Thus, the results of our analysis indicate that selecting points based on the image gradient can better extract motion information from the 2D US sequence.

### 4.4 Strain mapping validation

To the best of our knowledge, RBF-based methods and meshless methods in general have not been proposed to estimate the AAA wall strains. Contrary to mesh-based methods, meshless methods can adapt to sparse source points. To validate the strain estimation independently from the previously mentioned steps, we compared the RBF differentiation method to the mesh-based numerical approach, typical of FEM. The two approaches show similar convergence rates, RBF being slightly faster. Furthermore, we found a difference between the two methods of up to 20% of the FEM value in the high-strain zones. The tendency of RBF to provide higher strain and stress values with respect to the FEM is in agreement with what was previously found by Groth et al. (2022). Equivalent results were found by running the FEM with full integration elements. A possible strategy to reduce this distance could be to fine-tune the RBF parameters.

### 4.5 Wall strain patterns

Strains were computed from displacement fields by applying RBF interpolation and differentiation. Our results confirmed previous findings, revealing heterogeneous circumferential strains (Satriano et al., 2015; Cebull et al., 2019). A sector-wise analysis revealed a recurrent pattern in our data set. In most cases, the highest circumferential strains were found in the lateral walls, in line with the findings of Iffrig et al. (2019), obtained using 2D dynamic magnetic resonance imaging. In addition, we observed small or negative values of circumferential strain in the posterior wall, indicating the absence of circumferential stretch or a circumferential shortening in the posterior wall 7). In some cases, negative circumferential strains in the posterior wall were accompanied by positive radial strains (P2, P3, P5, and P6 in Figure 10). de Hoop et al. (2020) found similar results when mapping the aortic wall strains from 2D US in a phantom. In their study, a porcine aorta was embedded in an abdominal phantom and put in contact with a vertebral segment in order to mimic the boundary conditions of a human aorta. We hypothesize that the negative or small strains in the posterior wall could be associated with the motion constraint caused by the spine. However, a decrease in length in the circumferential direction that is not accompanied by a radial expansion would be more likely due to cyclic out-of-plane motion. Because the posterior wall experiences less in-plane motion due to the spine, as previously shown by Goergen et al. (2007), we also expect a higher impact of the out-of-plane motion (P4, P7, and P8 in Figure 10). Wall strain assessments from time-resolved 3D US revealed high circumferential strains happening in the posterior wall (Derwich et al., 2020), suggesting that the complex 3D motion of the AAA should be taken into account when interpreting 2D US-based strain maps. The described pattern did not appear in one patient, possibly due to the presence of intraluminal thrombus (P1 - Figure 7), which was previously found to have a large impact on the stress distribution in the wall (Lorandon et al., 2022).

### 4.6 Inter-cycle and inter-probe reproducibility

The inter-cycle reproducibility within the same sequence was assessed by comparing the strains in the peak systolic frames. Qualitative assessment on polar plots showed similar patterns for the different cycles within a single sequence. ICC values reveal good reliability of the measurements in most of the sequences acquired with the 2D probe, while relatively low inter-cycle reproducibility was observed for the bi-axial probe. Sources of inter-probe variability comprise the inter-acquisition transducer displacement and the lower spatial and temporal resolutions of the X6-1 probe with respect to the C5-1 probe, causing an increase in pixel size between 0.01 and 0.05 mm in both dimensions and a 50% decrease in the frame rate. The decreased temporal resolution in the X6-1 probe might also lead to missed maximum expansion peaks, resulting in higher inter-cycle variability.

### 4.7 Limitations and perspective work

The present study has some limitations. First, we validated the tracking algorithm, adapting a pipeline for myocardial sequence simulation Alessandrini et al. (2018). As in the myocardial tissue, the wall displacement field, computed via FEM simulation, is not coherent with the background displacement field, taken from the template sequence. Although a transition zone between the two scatter maps exists, there was still some discontinuity between the two motion fields because the background area is quite large compared to the wall area, which is a characteristic of AAA sequences. Although the SD algorithm only tracks the selected points, background motion influenced the tracking because of the image filtering performed as a pre-processing step for regularization. Therefore, a new validation pipeline should be proposed that imposes a coherent motion to the background motion of the template image. In addition, the impact of spatial and temporal resolution could be investigated by means of US simulations in order to better assess the robustness of the proposed method.

As a further limitation, we did not impose any mechanical equilibrium on the wall strain field. Therefore, we do not assume the wall to be a continuum. Enforcing equilibrium could provide a reconstruction of the strain field where the information from the B-mode images is missing. However, such an approach would also assume the absence of out-of-plane forces and surrounding tissues, leading to an erroneous reconstruction. Thus, a dedicated analysis should be performed to study the impact of these boundary conditions on the AAA wall mechanics. Adding a correction factor based on tracking in the longitudinal plane would help account for out-of-plane motion.

Last, our study is limited to a small cohort of patients and was sensitive to acquisition conditions, such as large motions and the presence of contrast medium in the blood. Clinical studies on larger cohorts of patients are needed to confirm our findings on the strain maps as well as to obtain a better insight into the reproducibility of strain measurements. The same probe intra-patient reproducibility should be assessed to better characterize the different factors affecting US-based mechanical estimations, including transducer placement, patient stillness, and sonographer level of expertise. In addition, the tracking algorithm could be made more robust to undesired motion by applying temporal smoothing and by re-initializing the point selection at the beginning of each cycle. By doing so, our method could generalize to less stable cycles, thus decreasing the need for sequence cropping and potentially broadening its applicability.

The efficiency of our method could be improved by better harnessing the sparse nature of the RBF differentiation for strain computation. In practice, instead of providing the full displacement and strain meshes as maps, as typically performed in the field (Mix et al., 2017; de Hoop et al., 2020; Zottola et al., 2022), we could output from our analysis only the clinically relevant information (e.g., the areas of high strain). Such an approach would speed up the computation and, thus, allow for a larger ROI selection. For example, the surrounding tissues could be included in the strain calculation to understand how they influence the AAA biomechanics. Because full automation is desired for reproducible and fast results, we aim to develop an automatic wall segmentation as well as an automatic sequence cropping and to study the impact of the initial wall delineation on the strain maps. Ultimately, wall strain could be estimated during the acquisition in order to have an overview of the AAA wall distensibility within the clinical assessment time.

## 5 Conclusion

In summary, we presented and demonstrated the feasibility of fast strain mapping in the AAA wall from 2D US B-mode cine-loop sequences. The presented method could provide 2D strain maps for various consecutive cycles within one sequence, being significantly faster than similar existing procedures. A strain pattern was observed in the majority of patients, suggesting biomechanical interaction with surrounding tissues and revealing the importance of out-of-plane motion. Thanks to the combination of the SD tracking algorithm and RBF differentiation for strain computation, our method was proven to be capable of adapting to the heterogeneous image quality of 2D US sequences, allowing the maximization of the extraction of information on the AAA wall motion from the 2D US cine-loops.

## Data Availability

The raw data supporting the conclusion of this article will be made available by the authors, without undue reservation.

## Abbreviations

2D US, two-dimensional ultrasound; 3D US, three-dimensional ultrasound; AAA, abdominal aortic aneurysm; AP, antero-posterior; BSC, backscattering coefficient; FEM, finite-element method; ICC, interclass correlation coefficient; RBF, radial basis function; RMSE, root mean square error; ROI, region of interest; SD, Sparse Demons.
