# Supplementary material for: Fast strain mapping in abdominal aortic aneurysm wall reveals heterogeneous patterns
Source: Front Physiol. 2023 Jun 8;14:1163204. doi: 10.3389/fphys.2023.1163204 (PMC10285457; doi:10.3389/fphys.2023.1163204)
Supplement: Supplementary file 1 [file DataSheet1.PDF]

Supplementary Material

1 SUPPLEMENTARY VIDEOS

Video 1: File ’StrainMap.mp4’ Circumferential strains in patient 8 mapped throughout the sequence, acquired with C5-1 probe. Eight cycles were analysed. The values are indicated in the colorscale on the right. The original sequence is zoomed in to allow for better visualisation.

Video 2: File ’simuSequenceVideo.avi’ Simulated 2D US cine-loop sequence from patient 3 template, consisting of one pressure cycle. The wall motion is imposed by a FEM simulation, while the background motion follows the first cycle of the template. The systolic peak in the simulation is synchronized with the corresponding peak in the first cycle of the template.

2 SUPPLEMENTARY FIGURES

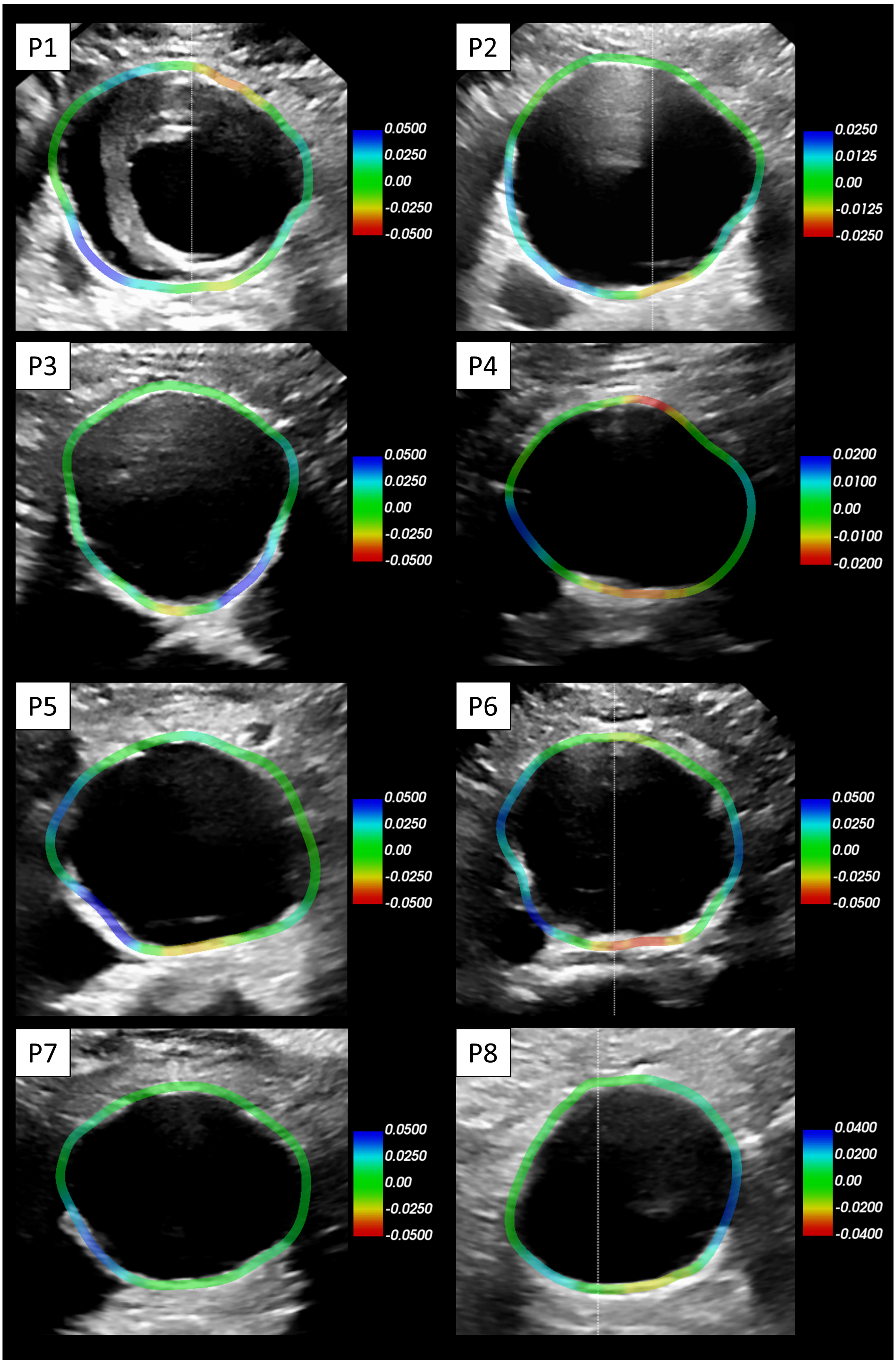

Figure S1. Circumferential strain maps depicted in the reference peak systolic frame of each patient acquired with the X6-1 probe. The color scale is adapted per patient based on the range of the strain in the sequence. Positive strains (blue) indicate circumferential stretch and negative (red) indicate circumferential shortening.

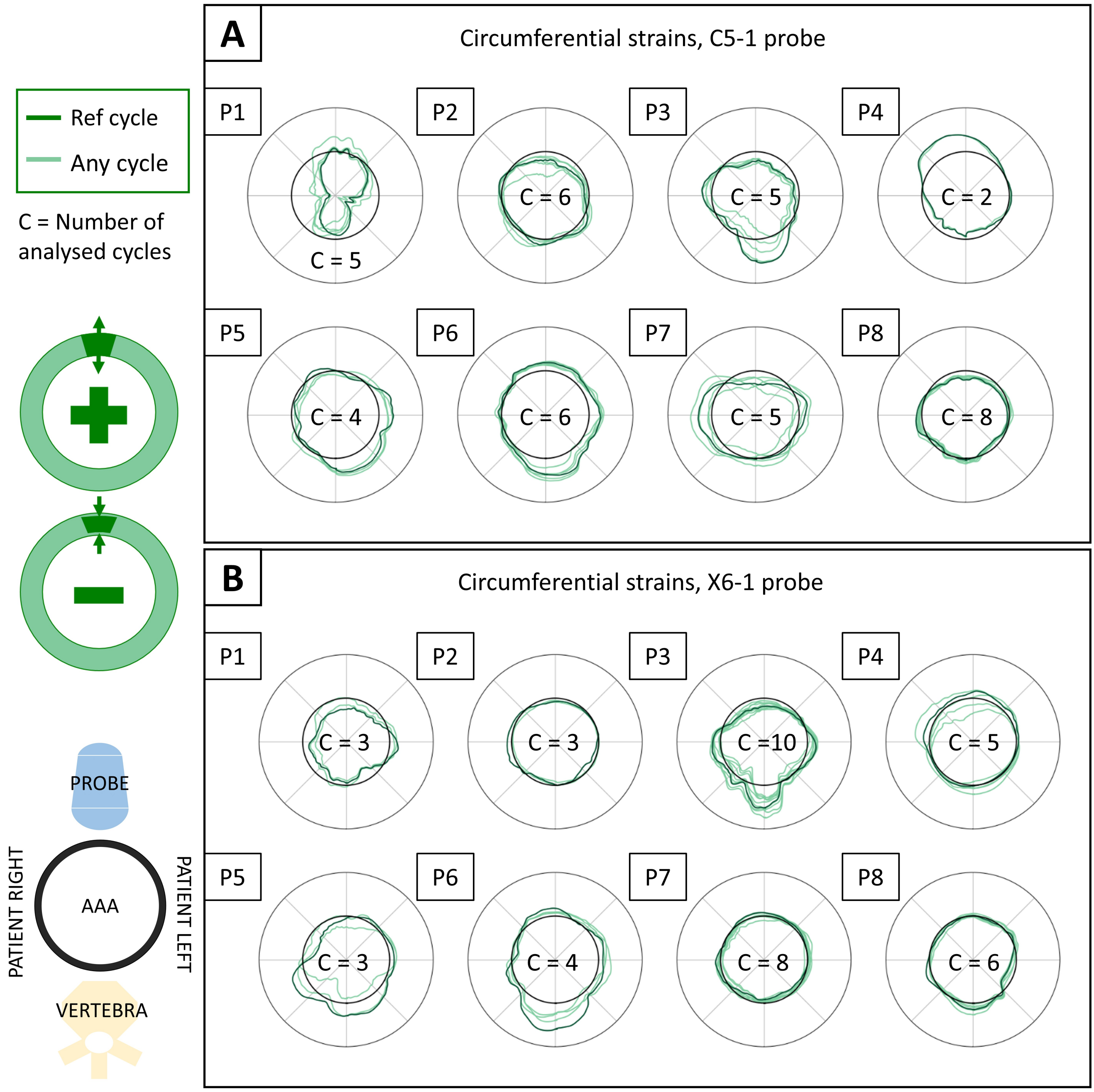

Figure S2. Radial strains in all patients and their inter-cycle reproducibility, reported for the C5-1 probe (Figure A) and for the X6-1 probe (Figure B). The polar plots depict the radial strains in the systolic peaks of each analysed cycle (in light red) and in the reference cycle (in dark red), averaged over the layers of the output grid. The representation is coherent with 2D US orientation. For each sequence, the number of analysed cycles is reported inside the polar plot. The strain values (radial coordinate in the plots) range from -0.01 to 0.01. The meaning of negative and positive values is illustrated in the scheme on the left.

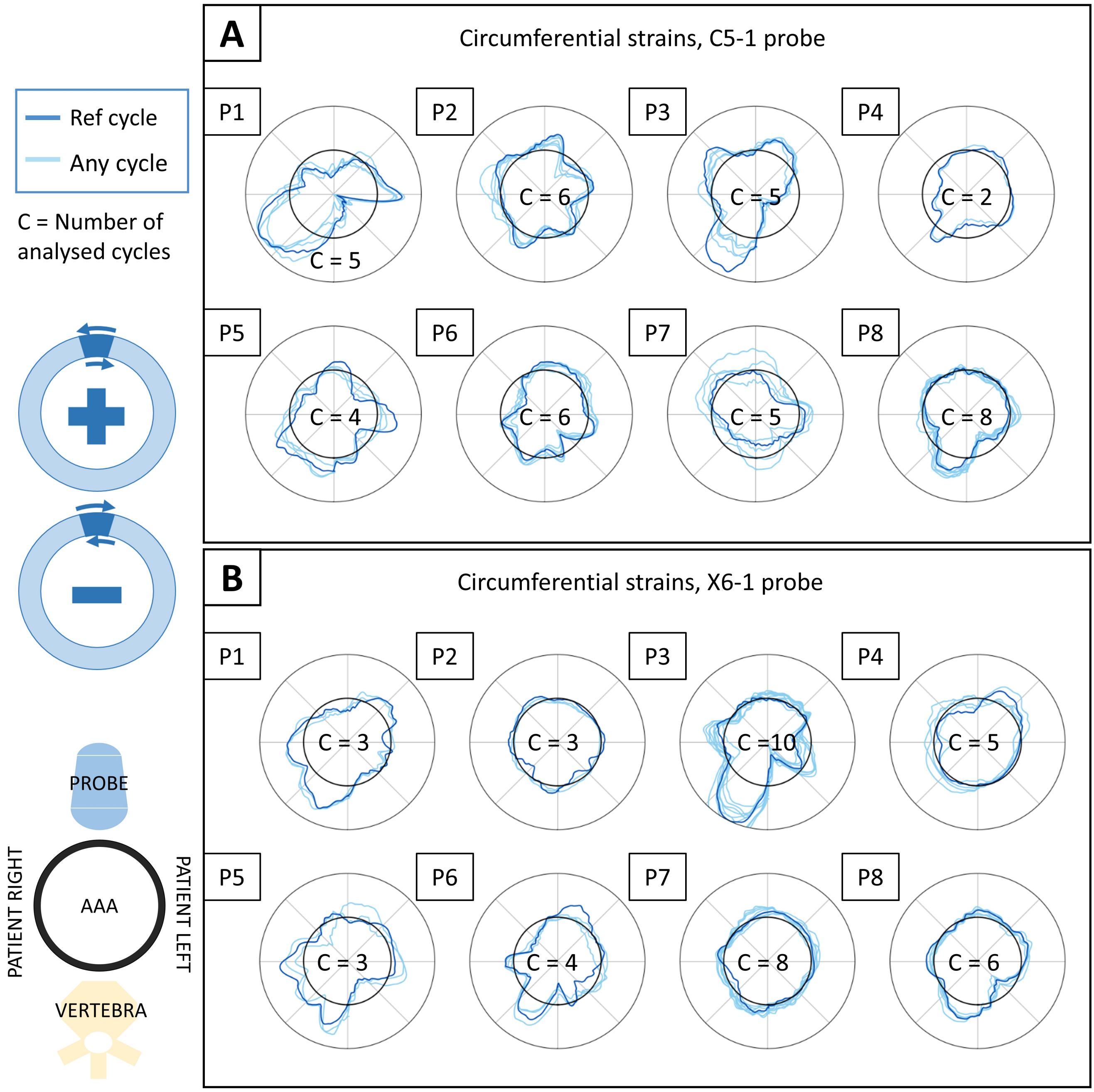

Figure S3. Shear strains in all patients and their inter-cycle reproducibility, reported for the C5-1 probe (Figure A) and for the X6-1 probe (Figure B). The polar plots depict the shear strains in the systolic peaks of each analysed cycle (in light red) and in the reference cycle (in dark red), averaged over the layers of the output grid. The representation is coherent with 2D US orientation. For each sequence, the number of analysed cycles is reported inside the polar plot. The strain values (radial coordinate in the plots) range from -0.01 to 0.01. The meaning of negative and positive values is illustrated in the scheme on the left.
